# The Portuguese version of the Screen for Disordered Eating: Validity and reliability in the perinatal period

**DOI:** 10.1192/j.eurpsy.2024.642

**Published:** 2024-08-27

**Authors:** A. T. Pereira, R. Lima, J. M. Pinto, D. Pereira, A. Macedo, C. Marques, A. I. Araújo, B. Barbosa, C. Pinto Gouveia

**Affiliations:** ^1^ Institute of Psychological Medicine; ^2^Faculty of Medicine, University of Coimbra, Coimbra, Portugal

## Abstract

**Introduction:**

Despite the increased knowledge about the prevalence and consequences of eating disorders (ED), they continue to be underdiagnosed and undertreated. Being more common in women of childbearing age, the perinatal period may play a decisive role in the incidence and course of these pathologies. The Screen for Disordered Eating (SDE) was developed for the screen of ED in primary care.

**Objectives:**

Our aim was to analyze the psychometric properties of the Portuguese Version of SDE in women during the perinatal period.

**Methods:**

Participants were 346 women with a mean age of 31.68 of years old (± 4.061; range: 18-42). 160 were pregnant (second or third trimester) and 186 were in the post-partum (mean baby´s age=4.37 months (± 2.87; range: 1-12). They answered an online survey including the Portuguese version of the SDE and of the Eating Disorder Examination – Questionnaire (EDE-Q-7).

**Results:**

Confirmatory Factor Analysis showed that the unidimensional model presented good fit indexes in pregnancy (), post-partum () and considering both – perinatal period (χ^2^/df=2.0335; RMSEA=.0547, p<.001; CFI=0.9976 TLI=0.9939, GFI=0.9906). The Cronbach’s alfa were ≥ 0.65. All the items contributed to the internal consistency and presented high internal validity. Pearson correlations between SDE and EDE-Q-7 total scores were significant (p<.001) positive and high in pregnancy (.639), postpartum (.583) and the perinatal period (.617).

**Conclusions:**

The Portuguese version of SDE has shown good validity (construct and concurrent) and internal consistency. As such, SDE might be a useful tool to screen ED in women during the perinatal period.

**Disclosure of Interest:**

None Declared

